# Short-Term Impact of Endoscopic Gastric Remodeling in Metabolic Dysfunction-Associated Steatotic Liver Disease: A Systematic Review and Meta-Analysis

**DOI:** 10.1007/s11695-026-08889-7

**Published:** 2026-08-11

**Authors:** Yusuf Kagzi, Abuzar Asif, Srinivas Reddy Puli

**Affiliations:** University of Illinois System, Peoria, IL USA

**Keywords:** Metabolic dysfunction-associated steatotic liver disease (MASLD), Endoscopic gastric remodeling, Endoscopic sleeve gastroplasty, Liver fibrosis, Weight loss, Insulin resistance

## Abstract

**Background:**

Metabolic dysfunction-associated steatotic liver disease (MASLD), a leading cause of chronic liver disease in the United States, is linked to obesity and insulin resistance. Endoscopic gastric remodeling (EGR) offers a safe and effective minimally invasive approach for MASLD. It is associated with improvements in noninvasive fibrosis test (NIT), liver enzymes, weight reduction, and metabolic parameters than bariatric surgery in the management of obesity. EGR includes three primary approaches: endoscopic sleeve gastroplasty (ESG), endoscopic gastric plication (EGP), and primary obesity surgery endoluminal (POSE).

**Aim:**

To evaluate the effects of EGR on MASLD-related liver outcomes and metabolic parameters.

**Methods:**

A systematic search of multiple electronic databases, including conference proceedings, was conducted from database inception through November 2025 to identify studies reporting hepatic or metabolic outcomes following EGR. Weighted mean differences and pooled proportions were calculated using a fixed-effects model. Heterogeneity was assessed using Cochran’s Q test. Primary outcomes included changes in noninvasive fibrosis indices: NAFLD Fibrosis Score (NFS), fibrosis-4 (FIB-4), and hepatic steatosis index (HSI). Secondary outcomes included liver enzymes, weight loss metrics, and glycemic parameters.

**Results:**

Six studies comprising 220 patients (mean age 45 years; 67.5% female; mean baseline body mass index 38.9 kg/m²) with follow-up ranging from 6 to 12 months. EGR was associated with significant improvements in liver fibrosis indices, including NFS (mean difference − 0.65; 95% CI − 0.89 to − 0.42), FIB-4 (mean difference − 0.07; 95% CI − 0.22 to 0.07), and HSI (reduced from 50.36 ± 3.00 to 42.66 ± 2.38). Reductions in liver enzymes were observed, with pooled decreases of 14.39 U/L in alanine aminotransferase and 6.58 U/L in aspartate aminotransferase. EGR resulted in weight loss, with a pooled percent excess weight loss of 47.80% and a percent total weight loss of 16.24%. Glycemic control improved, with a 0.33% reduction in hemoglobin A1c and a 62% decrease in insulin resistance. No significant publication bias was detected (Begg–Mazumdar Kendall’s tau b − 0.20, *p* = 0.48).

**Conclusions:**

Endoscopic gastric remodeling is a safe and effective minimally invasive therapy for MASLD. Further long-term studies are warranted to confirm the durability of these benefits.

## Introduction

Metabolic dysfunction-associated steatotic liver disease (MASLD), formerly known as nonalcoholic fatty liver disease (NAFLD), has been recognized as the leading cause of chronic liver disease worldwide. In the United States, MASLD affects over 38% of the population, marking a 50% increase over the past three decades. Its prevalence is even higher, ranging from 55% to 70% among individuals with type 2 diabetes mellitus (T2DM) [1, 2]. The shift in terminology from NAFLD to MASLD reflects a more accurate pathophysiological understanding, focusing on the presence of metabolic dysfunction rather than excluding other causes such as alcohol or viral hepatitis. In patients with obesity and metabolic syndrome, the risk of progression to advanced liver disease, including fibrosis, cirrhosis, and hepatocellular carcinoma, exceeds 50%, compared to approximately 25% in the general population [3, 4].

Diagnosis of MASLD requires evidence of hepatic steatosis along with overweight/obesity, T2DM, or other metabolic risk factors such as elevated waist circumference, hypertension, or prediabetes even in individuals with normal body weight [1, 2]. Staging of MASLD depends on identifying hepatic steatosis through imaging, serum biomarkers, or histological assessment. Although liver biopsy remains the gold standard for evaluating disease severity and fibrosis, its invasiveness limits its routine use in clinical practice [5, 6]. As a result, noninvasive alternatives have gained more prominence. Imaging-based noninvasive assessment with transient elastography (FibroScan) or shear wave elastography which measures liver stiffness and the controlled attenuation parameter (CAP), offers a more practical and reliable assessment [7, 8]. In addition, noninvasive scoring systems such as the NAFLD Fibrosis Score (NFS), fibrosis-4 Index (FIB-4), and hepatic steatosis index (HSI) are useful for risk stratification and for ruling out advanced fibrosis. Serum biomarkers including alanine aminotransferase (ALT), aspartate aminotransferase (AST), glycated hemoglobin (HbA1c), and homeostatic model assessment for insulin resistance (HOMA-IR) further aid in evaluating metabolic status and liver function [8, 9].

Despite advancements in diagnostic modalities, effective therapeutic options for MASLD remain limited. Lifestyle modification, including a hypocaloric Mediterranean-style diet rich in unsaturated fats and low in processed sugars and red meat, along with reduction in alcohol intake, remains the cornerstone of MASLD management with improvement in liver-related outcomes (≥ 5% weight loss reducing steatosis, ≥ 7% can resolve steatohepatitis, and ≥ 10% may reverse fibrosis) [10, 11].

Pharmacologic treatment options for MASLD have evolved rapidly, with current evidence derived from multiple randomized controlled trials. A review of 77 trials involving various drug classes like antioxidants, bile acids, and thiazolidinediones have shown no clear benefit on mortality or serious adverse events [12]. Glucagon-like peptide-1 receptor agonists (GLP-1RAs) reduce metabolic parameters, liver fat, and inflammation while aiding weight loss but may cause gastrointestinal side effects. Newer therapies such as resmetirom, a selective thyroid hormone receptor beta agonist, show efficacy in early stages of liver fibrosis and recently clarified the clinical use in appropriate patients with ongoing studies. Other agents like SGLT2 inhibitors, statins, and mRNA-based therapies are under investigation for their potential in MASLD treatment [13]. While lifestyle changes and pharmacologic treatments are preferred options, they often fail to achieve lasting weight loss or metabolic benefits. In contrast, bariatric surgery including Roux-en-Y gastric bypass (RYGB), laparoscopic sleeve gastrectomy (LSG), and Lap Band is more effective but is also invasive, costly, and less accessible, with a 0.3% postoperative mortality rate, failure rates of 15–50%, and weight regain observed in up to 70% of patients by six years [14–18].

The American Society for Gastrointestinal Endoscopy (ASGE) recently adopted endoscopic gastric remodeling (EGR) techniques including endoscopic sleeve gastroplasty (ESG), endoscopic gastric plication (EGP), and primary obesity surgery endoluminal (POSE). These procedures achieve gastric volume reduction and foregut remodeling, offering minimally invasive, flexible alternatives for weight loss and metabolic improvement even for patients with lower BMIs (≥ 30 kg/m² or less with comorbidities). ESG reduces stomach volume by about 80% through full-thickness sutures along the greater curvature, creating a tubular gastric configuration, delaying gastric emptying, and increasing satiety. Similarly, EGP decreases stomach size by creating internal folds via endoscopic suturing plication techniques, while POSE uses transmural tissue anchors primarily in the gastric fundus and body to limit fundal accommodation and slow gastric emptying [19, 20].

Given the growing interest in less invasive therapies for MASLD, evaluating the efficacy of EGR and its benefits beyond weight reduction is essential. This systematic review and meta-analysis aim to assess the impact of EGR on liver-specific outcomes such as fibrosis indices, and liver enzymes (ALT, AST) as well as metabolic outcomes like weight loss and glycemic control in MASLD patients.

## Materials and Methods

### Search Methodology

A comprehensive literature search was conducted using MEDLINE through PubMed, Embase, Cochrane Library (Central and Database of Systematic Reviews), Google Scholar, Scopus, and Web of Science from database inception through November 2025. The search followed PRISMA (Preferred Reporting Items for Systematic Reviews and Meta-Analyses) guidelines and included relevant conference proceedings. The search terms included: “Endoscopic gastric remodeling” OR “EGR”, “Endoscopic sleeve gastroplasty” OR “ESG”, “Endoscopic gastric plication”, “Primary obesity surgery endoluminal” OR “POSE”, “MASLD” OR “Metabolic dysfunction–associated steatotic liver disease” OR “MAFLD” OR “Metabolic dysfunction-associated fatty liver disease”, “NAFLD” OR “Nonalcoholic fatty liver disease”, “hepatic steatosis”, and “liver fibrosis”. Reference lists of retrieved articles were manually screened for additional eligible studies. Duplicate and overlapping data were excluded after thorough cross-checking.

### Study Eligibility

Studies were eligible for inclusion if they reported outcomes related to liver-specific or metabolic parameters following EGR procedures in adult human subjects. Eligible studies had to report at least one relevant outcome measure. Articles were excluded if they were not in the English language. Studies in animal models, editorials, and comments were excluded. Two authors (AA and SP) reviewed the full-text articles independently.

### Data Extraction and Quality Assessment

Two authors (AA and YK) independently extracted data using a standardized form. Extracted variables included: (a) study characteristics (first author, year of publication, study design, country); (b) population demographics (sample size, baseline body mass index); (c) intervention details (type of EGR procedure, follow-up duration); and (d) outcomes including liver-specific indices (NFS, FIB-4, HSI), component laboratory and clinical variables used in these indices where available (AST, ALT, age, BMI, diabetes status, platelet count, and albumin), weight loss outcomes, and metabolic parameters as mentioned below. Discrepancies were resolved by consensus.

### Outcomes Evaluated

The primary outcomes assessed were changes in NFS, FIB-4 score, and HSI following EGR. Secondary outcomes included improvement in % total weight loss (TWL), % excess weight loss (EWL), body mass index (BMI), HbA1c, and insulin resistance (HOMA-IR), liver enzymes (ALT and AST), and procedure-related adverse events.

Weight-loss outcomes included %TWL, calculated as [(baseline weight − postoperative weight) / baseline weight] × 100, and %EWL, calculated as [(initial weight − postoperative weight) / (initial weight − ideal weight)] × 100, where ideal weight corresponds to a BMI of 25 kg/m².

### Statistical Analysis

This meta-analysis was performed by calculating weighted pooled effects. Continuous outcomes were summarized using weighted mean differences (WMDs). Individual study proportions were transformed using the Freeman–Tukey variant of the arcsine square-root transformation. The pooled proportion was calculated as the back-transformation of the weighted mean of the transformed proportions using inverse arcsine variance weights for the fixed-effects model and DerSimonian–Laird weights for the random-effects model. Weighted mean differences were pooled using inverse variance weighting [21, 22].

Heterogeneity among studies was assessed using Cochran’s Q test based on inverse variance weights and quantified using the I² statistic. I² values of 0–30% were considered low heterogeneity, 30–60% moderate heterogeneity, 60–75% substantial heterogeneity, and > 75% considerable heterogeneity [23]. A *p*-value > 0.10 rejects the null hypothesis that the studies are heterogeneous. Forest plots were drawn to show the point estimates in each study in relation to the summary of the pooled estimate. The width of point estimates in the Forest plots indicates the weight assigned to that study. The Egger and Begg-Mazumdar bias indicators assessed the effects of publication and selection bias on summary estimates [24, 25]. Funnel plots were constructed to assess potential publication bias [26, 27]. Microsoft Excel 19 was the software used to perform statistical calculations for this meta-analysis. Statistical analyses were performed using Microsoft Excel 2019 (Microsoft Corporation, Redmond, WA, USA) with inverse-variance fixed-effects. This systematic review and meta-analysis was not registered in PROSPERO or other systematic review registry.

## Results

### Study Selection and Characteristics

**Fig. 1:**
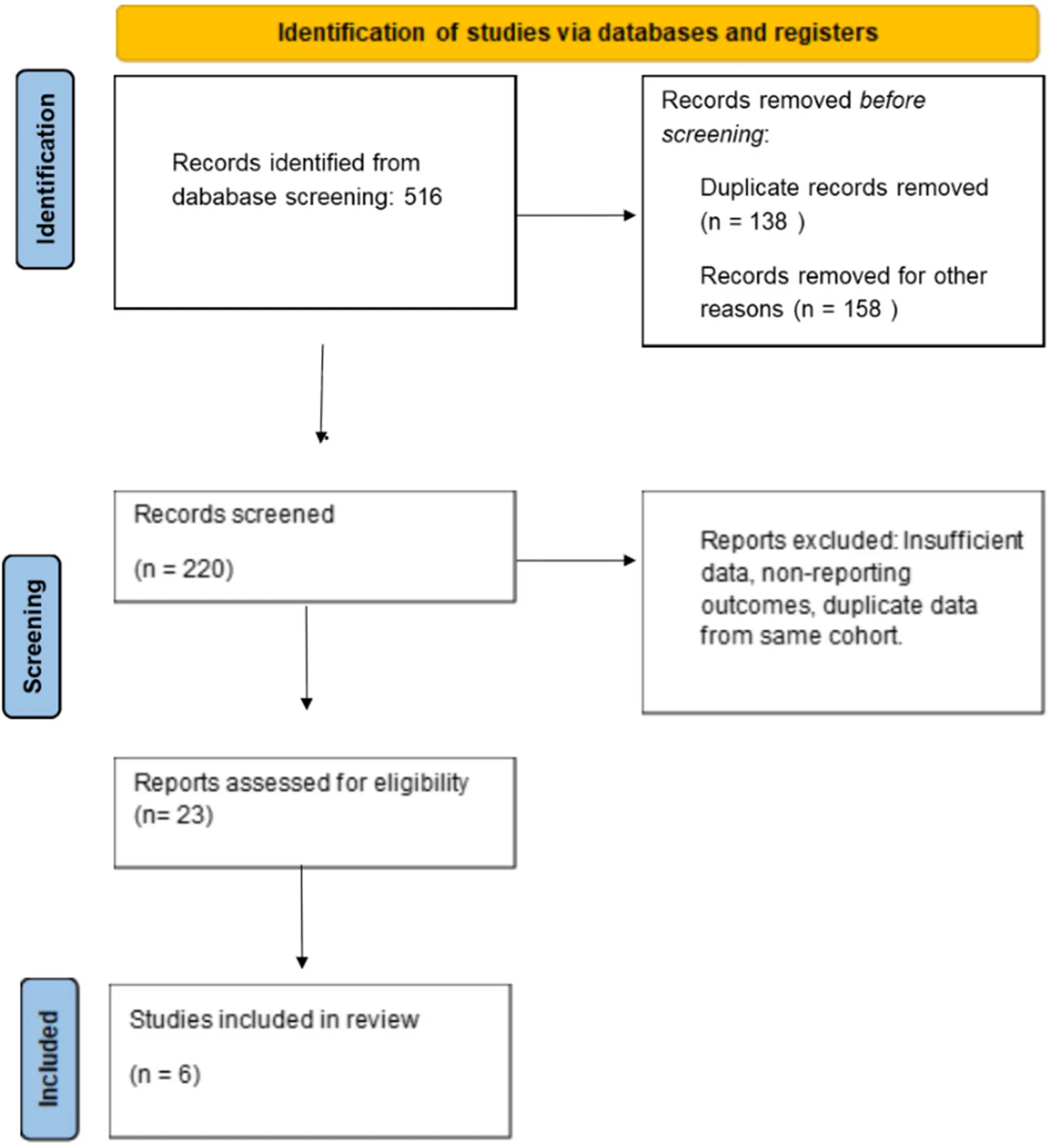
PRISMA flow diagram depicting the selection process of studies included in the meta-analysis

**Fig. 2:**
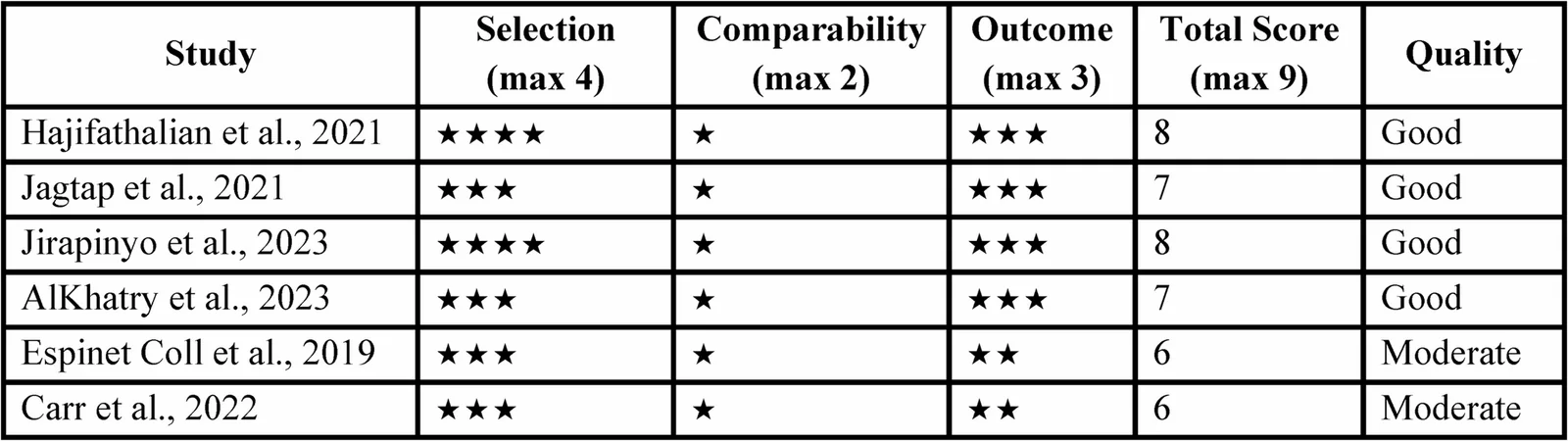
Quality assessment of included studies using the Newcastle–Ottawa scale

An initial search yielded 516 studies, of which 23 full-text articles were reviewed in detail. A total of 6 studies met the inclusion criteria [19, 28–32]. The PRISMA flow diagram outlining the selection process is provided in Fig. 1. The quality of studies was good, as evaluated using the modified Newcastle-Ottawa scale (Fig. 2). All the pooled estimates given are estimates calculated using the fixed-effects model. The estimates calculated using fixed- and random-effects models were similar. The agreement between reviewers was 1.0, as measured by Cohen’s κ.

Among the 240 included patients, ESG was performed in 175 (72.9%), EGP in 45 (18.8%), and POSE in 20 (8.3%). The mean age across studies was 45 ± 5.4 years, and 67.5% of the participants were female. The average baseline BMI was 38.88 ± 1.59 kg/m². The follow-up duration ranged from 6 to 12 months across studies, and the most performed procedure was ESG.

## Primary Outcomes

### Improvement in NFS

**Fig. 3:**
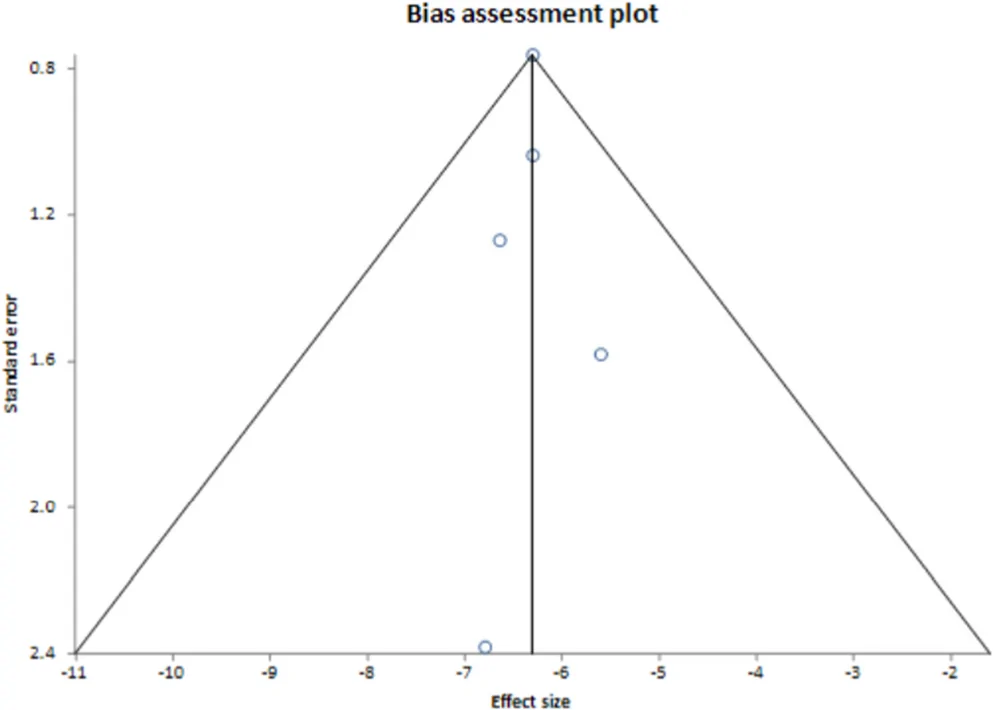
Forest plot showing individual study rates and weighted pooled rate of NAFLD fibrosis score following EGR

**Fig. 4:**
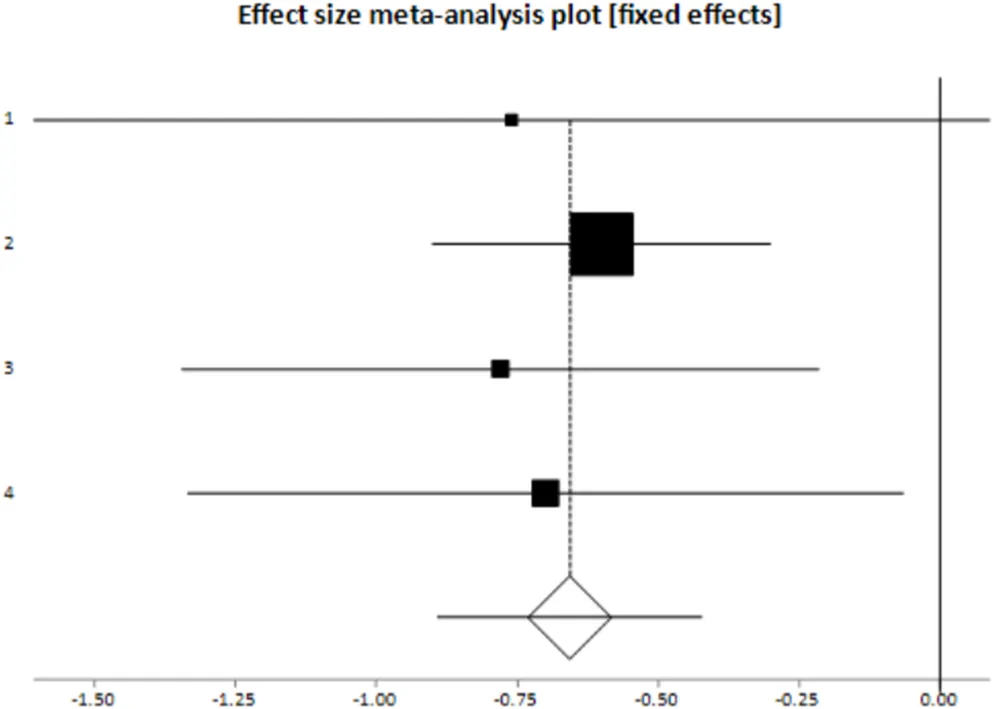
Funnel plot providing potential publication bias within the meta-analysis using funnel plot asymmetry and regression-based bias indicators

EGR showed improvement in the NFS with a pooled weighted mean difference of -0.65 (95% CI: -0.89 to -0.42). The mean score decreased from − 0.63 ± 1.10 preoperatively to -1.58 ± 0.74 postoperatively. There was no significant heterogeneity with an I² = 0%. A forest plot displaying individual study estimates and the pooled estimate is shown in Fig. 3. The Begg-Mazumdar bias indicator yielded a Kendall’s tau b value of − 0.2 (*p* = 0.48), indicating no publication bias. A funnel plot assessing publication bias is shown in Fig. 4.

### Improvement in FIB-4

Following EGR, the FIB-4 score showed a reduction with a pooled weighted mean difference of − 0.07 (95% CI: − 0.22 to 0.07). The mean score reduced from 1.46 ± 0.87 preoperatively to 1.34 ± 0.56 postoperatively.

### Improvement in HSI

The mean HSI following EGR decreased from 50.37 ± 3.00 preoperatively to 42.66 ± 2.39 postoperatively.

## Secondary Outcomes

### Reduction in Liver Enzymes

ALT levels decreased from a preoperative mean of 40.24 ± 10.14 U/L to 31.03 ± 9.31 U/L postoperatively, with a pooled weighted mean difference of − 14.39 (95% CI: − 18.94 to − 9.85). Similarly, AST levels declined from 42.36 ± 22.17 U/L to 23.30 ± 1.15 U/L, corresponding to a pooled weighted mean difference of − 6.58 (95% CI: − 8.39 to − 4.77).

### Weight Loss Outcomes

Weight loss outcomes were assessed using percent total weight loss (TWL), percent excess weight loss (EWL), and change in BMI to evaluate the effectiveness of EGR. The mean %TWL was 16.25% (95% Cl: 14.99% to 17.51%), while the mean %EWL was 47.80% (95% Cl: 41.03% to 54.57%). In addition, BMI decreased from a baseline mean of 38.88 ± 1.59 kg/m² to 31.45 ± 1.32 kg/m² following the intervention, corresponding to a pooled weighted mean difference of − 6.30 kg/m² (95% CI: −7.30 to − 5.31).

### Glycemic Control and Insulin Resistance

HbA1c levels decreased from 6.18 ± 1.04% preoperatively to 5.73 ± 0.64% postoperatively, with a pooled weighted mean difference of − 0.33 (95% CI: − 0.44 to − 0.21). Insulin resistance, measured by the HOMA-IR, also declined from 8.30 ± 3.91 pre-procedure to 3.10 ± 1.37 post-procedure.

### Adverse Event Rate

**Fig. 5:**
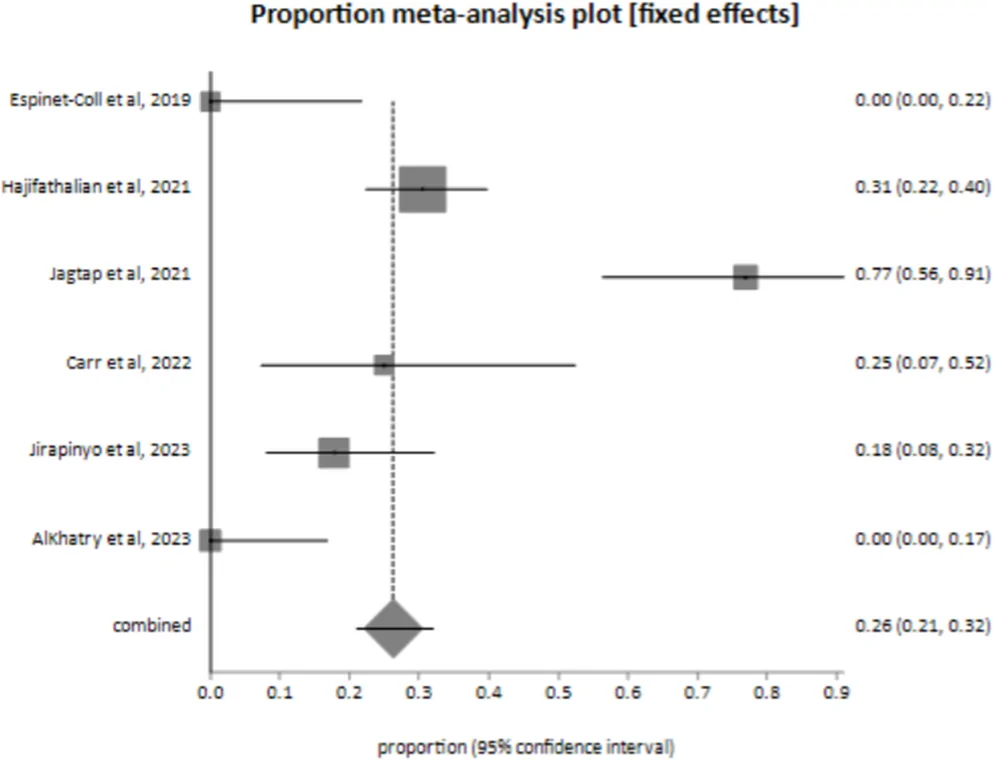
Forest plot showing individual study rates and weighted pooled rate of overall adverse events following EGR using fixed effects model

Overall pooled adverse event rate reported in our study was 26.3% (95% CI: 20.99% to 32.04%). A forest plot summarizing individual and pooled estimates is shown in Fig. 5. Most adverse events were mild and self-limited, including nausea, vomiting, and abdominal pain (*n* = 68). Clinically significant adverse events occurred in three patients (1.4%) include perigastric leak (*n* = 1), gastric perforation (*n* = 1), and esophageal tear (*n* = 1). No procedure-related deaths were reported.

## Discussion

Our analysis reports on the potential role for ESG, EGP, and the POSE procedure under the unified category of EGR as a therapeutic option beyond weight reduction in improving short-term hepatic and metabolic outcomes in patients with MASLD [19, 20, 33]. Validated non-invasive fibrosis scores such as FIB-4, NFS, and HSI were utilized to assess improvements in NITs associated with fibrosis risk [34, 35]. Being a surrogate marker, these results should not be interpreted as direct evidence of fibrosis regression or histologic improvement.

The NFS incorporates age, BMI, diabetes status, AST/ALT ratio, platelet count, and albumin levels to estimate the risk of advanced liver fibrosis. Scores below − 1.46 help rule out advanced fibrosis (negative predictive value ~ 88%), while scores above 0.68 may indicate a higher likelihood of fibrosis. In our analysis, EGR reported a mean reduction of approximately 0.95 points in the NFS, representing a 67% shift toward a lower fibrosis risk category. These findings suggest a favorable shift in patients with MASLD from an indeterminate or high-risk group to a low-risk group after EGR in NITs [8, 36, 37]. While data on lifestyle-induced changes in NFS are limited, one study reported a small improvement of 0.5 points (from − 1.3 to − 1.8) after 1 year following lifestyle modifications [38]. In comparison, a large national cohort study by Seyedi et al. reported a decrease in NFS from − 0.3 to − 0.8 over two years following bariatric surgery [39]. Similarly, a long-term study by Sandvik et al. observed a 0.4 point reduction in NFS (from − 1.3 to − 1.7) over 11 years in patients undergoing Roux-en-Y gastric bypass (RYGB) [40]. Despite being less invasive, EGR achieved comparable reductions in NFS, suggesting a lower-risk alternative for patients with obesity and MASLD who are either not candidates for or prefer to avoid surgery.

Similarly, FIB-4 index is a widely used, non-invasive tool for assessing liver fibrosis, calculated using age, AST, ALT, and platelet count. A score below 1.45 rules out advanced fibrosis with a negative predictive value of 90%, while a score above 3.25 predicts advanced fibrosis with a specificity of 97% [8, 41–43]. In our analysis, EGR led to a 8% improvement in the FIB-4 score, with the mean score decreasing from 1.4 to 1.3 postoperatively. The modest change in FIB-4 scores observed in our cohort post-EGR may be due to several factors. First, FIB-4 is heavily influenced by age and laboratory parameters, which may not change substantially in a short postoperative timeframe or in early-stage fibrosis [44, 45]. Interpretation of FIB-4 should also be made cautiously, as previous studies have shown that FIB-4 may underperform in patients younger than 35 years. Because individual patient-level age distributions were not available, we were unable to determine how many participants were younger than 35 years [44]. Second, as a surrogate marker, FIB-4 may lack the sensitivity to detect subtle improvements in fibrosis that could be captured by imaging or histological assessment [44, 46]. A previous study evaluating lifestyle modifications over one year also observed a small reduction in the FIB-4 score, from 1.15 to 1.04 (mean change − 0.11) [38]. Comparatively, Seyedi et al. reported that the FIB-4 index initially increased at three months following bariatric surgery, then declined within the first year to 0.9, but showed a slight rise to 1.7 at two years indicating an inconsistent trend with minimal long-term improvement [39]. Similarly, Sandvik et al. reported outcomes after RYGB found only a slight change in the median FIB-4 score, from 0.81 to 0.89 [40]. Although the magnitude of improvement in FIB-4 scores following EGR was modest, the downward trend in FIB-4 scores following EGR aligns with patterns seen in lifestyle and surgical cohorts. However, the true effectiveness of FIB-4 as a response marker remains uncertain [47].

Lastly, the HSI, originally proposed by Lee et al., showed a mean reduction of approximately 8 points following EGR, corresponding to a 15% decrease. The HSI is calculated based on sex, BMI, AST, ALT, and the presence of T2DM. An HSI score > 36 suggests hepatic steatosis with a specificity of up to 92%, while a score < 30 rules it out with a sensitivity of up to 93% [48]. Although data comparing HSI reduction following bariatric surgery are limited, a Korean study reported decreases from 50 to 40 in the LSG group (20% reduction) and from 52 to 41 in the RYGB group (21% reduction) over a 6-month period [49]. In our analysis, EGR achieved a comparable reduction in HSI.

Reduction in liver enzymes, particularly ALT and AST, further supports the improvement in hepatic inflammation and liver function following EGR. These enzymes serve as surrogate markers for liver injury [50]. Our study demonstrated reductions of approximately 14 U/L in ALT and 6 U/L in AST post-EGR, which are comparable to reductions reported after bariatric surgery and lifestyle interventions. A national cohort study of bariatric surgery patients observed decreases of 13 U/L in ALT and 6 U/L in AST over two years, while lifestyle modifications led to reductions of 16 U/L in ALT and 8 U/L in AST [38, 39].

Additionally, EGR reported favorable weight loss outcomes using standardized metrics per American Society for Metabolic and Bariatric Surgeons. These include %TWL, calculated as [(initial weight – postoperative weight) / initial weight] × 100, and %EWL [(initial weight – postoperative weight) / (initial weight – ideal weight)], where ideal weight corresponds to a BMI of 25 kg/m² [51]. Post-EGR, the mean reductions in %TWL and % EWL were 16% and 47%, respectively, at 12-month follow-up. This degree of weight reduction exceeds the 7–10% threshold associated with histological improvement in MASLD and compares favorably with results from prior studies on ESG (17% TWL, 63% EWL) and the POSE procedure (13% TWL, 42% EWL) [52–54]. Liver fibrosis regression is associated with achieving ≥ 10% TWL; our findings reinforce the therapeutic potential of EGR not only for weight loss but also for improving hepatic outcomes [38]. This is particularly relevant, as lifestyle interventions alone often fail to achieve sufficient weight reduction, as highlighted by a meta-analysis showing that more than half of patients undergoing lifestyle modification did not reach the 7% TWL target [52].

In this context, the resolution of hepatic steatosis becomes even more critical, as it may not only improve liver health but also help prevent the onset of hyperglycemia. This is particularly relevant given the high prevalence of type 2 diabetes and obesity, which are well-established independent risk factors for MASLD [55, 56]. In our study, BMI decreased by over 6 kg/m² following EGR, which aligns with previous reports of BMI reductions ranging from 5% to 6% in patients undergoing endoscopic bariatric procedures [52–54]. Importantly, the metabolic benefits extended beyond weight loss, with improvements observed in insulin sensitivity with a 62% decline in HOMA-IR. These metabolic improvements are meaningful given the high prevalence of T2DM and insulin resistance in individuals with MASLD. Notably, all included studies reported reductions in baseline HbA1c, and only one study had a mean post-procedure HbA1c above 6.5% [30]. A reduction in HbA1c has been shown to increase the probability of fibrosis regression by up to 60% [57]. In contrast, lifestyle interventions often produce lower glycemic improvements, typically less than 1% reduction in HbA1c [38]. Taken together, the observed improvements following EGR serve as an effective therapeutic strategy in the management of MASLD.

Moreover, EGR was associated with an acceptable safety profile. Most adverse events were self-limited and managed conservatively, consistent with the minimally invasive nature of these procedures. Although serious adverse events were reported infrequently, no procedure-related mortality occurred. These findings are in line with prior studies of EGR and support the role of EGR as a less invasive alternative to bariatric surgery for selected patients [19, 28–32].

The therapeutic landscape for MASLD remains limited, with few pharmacological agents approved, and lifestyle interventions often fall short in long-term adherence. EGR offers a minimally invasive, repeatable, and cost-effective alternative that addresses multiple facets of the disease. Our findings are consistent with previously published analyses evaluating endoscopic bariatric therapies in MASLD. Nunes et al. conducted a meta-analysis of four studies including 175 patients undergoing ESG and reported improvements in HSI (− 4.85), NFS (− 0.50), ALT (− 6.32 U/L), HbA1c (− 0.51%), and weight loss outcomes (17.28% TWL and 47.97% EWL). In comparison, our analysis demonstrated similar or greater improvements, including reductions in NFS (− 0.65), ALT (− 14.39 U/L), HSI (50.36 to 42.66), with comparable weight loss (16.24% TWL and 47.80% EWL). Unlike prior studies, our analysis evaluates multiple EGR modalities in patients with MASLD. Its ability to induce sustained weight loss, improve insulin resistance, and reduce hepatic steatosis and fibrosis risk positions EGR as a compelling option in MASLD management, particularly for patients who are poor surgical candidates or who prefer endoscopic over surgical interventions [19, 20, 33].

EGR is adaptable over time, allowing for supplemental interventions as needed. However, the success of these techniques depends not only on mechanical restriction but also on modulating the complex neurohormonal responses that govern appetite and metabolism. An additional area of interest is the combination of EGR with anti-obesity pharmacotherapy, particularly glucagon-like peptide-1 receptor agonists and incretin-based therapies. The combination may provide synergistic benefits through gastric restriction, appetite regulation, and metabolic improvement. Future studies should evaluate whether combined treatment strategies can further enhance hepatic and metabolic outcomes [58, 59].

Our study has a few limitations. First, like many previous studies on endoscopic interventions for MASLD, it did not include histologic confirmation of steatosis or fibrosis, which remains the gold standard for diagnosis and staging of MASLD. Although ESG, EGP, and POSE are categorized as EGR, these procedures differ in their mechanisms of action. ESG induces gastric tubularization and volume restriction through full-thickness suturing, whereas EGP utilizes endoscopic plications and POSE relies on tissue anchors that reduce gastric or fundal accommodation. These physiological differences may influence metabolic and hepatic responses. However, due to the limited number of available studies, procedure-specific analyses were not feasible. Pooled findings should be interpreted as the overall impact of EGR rather than the efficacy of any single technique. Additionally, comparisons between EGR outcomes and those reported for bariatric surgery or lifestyle interventions should be interpreted cautiously, as these represent indirect comparisons across different studies. Further, primary hepatic outcomes were based on NIT, which are validated for risk stratification but have not been fully established as robust markers; therefore, improvements should be interpreted as changes in surrogate measures rather than direct evidence of fibrosis regression. In our analysis, some studies had small sample sizes and single-center designs, limiting the generalizability of our findings. The absence of a control group, such as a lifestyle or surgical intervention arm, limits the ability to directly compare the effectiveness of endoscopic therapies with other established treatment modalities.

The management of MASLD continues to evolve with advances pharmacologic therapies, EGR, bariatric surgery, and lifestyle interventions often fall short in long-term adherence. EGR had an acceptable safety profile across the included studies. EGR may serve as a minimally invasive, repeatable, and cost effective option that address multiple facets of the disease. It may also serve as part of a multidisciplinary treatment strategy, with the potential to be combined with pharmacologic therapies or supplemented by additional endoscopic or surgical interventions when clinically indicated. As evidence continues to emerge, future studies with longer follow-up and histologic validation are needed to further define the role of endoscopic bariatric interventions in the treatment algorithm for MASLD.

## Conclusions

EGR is associated with favorable short term inprovement in NIT, weight loss, and metabolic parameters in patients with MASLD, with a favorable safety profile. According to current evidence, bariatric and metabolic surgery remains the most effective intervention for achieving sustained weight loss and metabolic improvement. EGR is a minimally invasive option, particularly for patients who are poor surgical candidates.

## Data Availability

No datasets were generated or analysed during the current study.
